# Supplementary material for: Modeling glioblastoma heterogeneity as a dynamic network of cell states
Source: Mol Syst Biol. 2021 Sep 16;17(9):e10105. doi: 10.15252/msb.202010105 (PMC8444284; doi:10.15252/msb.202010105)
Supplement: Supplementary file 5 — Source Data for Figure 3 [file MSB-17-e10105-s001.zip › Figure3A_sourcedata/GSEA_3065/hallmarks_state3.GseaPreranked.1623416274744/HALLMARK_APICAL_SURFACE.html]

Details for gene set HALLMARK\_APICAL\_SURFACE[GSEA]

|  || Dataset | state3 |
| Phenotype | NoPhenotypeAvailable |
| Upregulated in class | na\_pos |
| GeneSet | HALLMARK\_APICAL\_SURFACE |
| Enrichment Score (ES) | 0.4702365 |
| Normalized Enrichment Score (NES) | 1.3028493 |
| Nominal p-value | 0.15071283 |
| FDR q-value | 0.12813437 |
| FWER p-Value | 0.696 |
Table: GSEA Results Summary

  

Fig 1: Enrichment plot: HALLMARK\_APICAL\_SURFACE      
 Profile of the Running ES Score & Positions of GeneSet Members on the Rank Ordered List

  

| PROBE | GENE SYMBOL | GENE\_TITLE | RANK IN GENE LIST | RANK METRIC SCORE | RUNNING ES | CORE ENRICHMENT || 1 | APP |  |  | 49 | 0.437 | 0.1820 | Yes |
| 2 | DCBLD2 |  |  | 243 | 0.265 | 0.2739 | Yes |
| 3 | THY1 |  |  | 326 | 0.236 | 0.3660 | Yes |
| 4 | BRCA1 |  |  | 339 | 0.232 | 0.4642 | Yes |
| 5 | CX3CL1 |  |  | 1077 | 0.133 | 0.4367 | Yes |
| 6 | SULF2 |  |  | 1232 | 0.119 | 0.4702 | Yes |
| 7 | EPHB4 |  |  | 1787 | 0.087 | 0.4443 | No |
| 8 | ADAM10 |  |  | 2347 | 0.063 | 0.4073 | No |
| 9 | B4GALT1 |  |  | 3043 | 0.040 | 0.3450 | No |
| 10 | ADIPOR2 |  |  | 3051 | 0.040 | 0.3613 | No |
| 11 | SRPX |  |  | 3518 | 0.028 | 0.3199 | No |
| 12 | TMEM8B |  |  | 3656 | 0.024 | 0.3145 | No |
| 13 | PLAUR |  |  | 4075 | 0.013 | 0.2723 | No |
| 14 | GAS1 |  |  | 4210 | 0.009 | 0.2610 | No |
| 15 | NCOA6 |  |  | 5253 | -0.017 | 0.1489 | No |
| 16 | GSTM3 |  |  | 5615 | -0.028 | 0.1195 | No |
| 17 | CRYBG1 |  |  | 5745 | -0.031 | 0.1181 | No |
| 18 | SHROOM2 |  |  | 6544 | -0.058 | 0.0517 | No |
| 19 | FLOT2 |  |  | 7064 | -0.085 | 0.0287 | No |
| 20 | HSPB1 |  |  | 8572 | -0.383 | 0.0205 | No |
Table: GSEA details [plain text format]

  

Fig 2: HALLMARK\_APICAL\_SURFACE: Random ES distribution      
 Gene set null distribution of ES for **HALLMARK\_APICAL\_SURFACE**
